# *Orientia*, *Rickettsia*, and the microbiome in rodent attached chiggers in North Carolina, USA

**DOI:** 10.1371/journal.pone.0311698

**Published:** 2024-12-05

**Authors:** Elise A. Richardson, Reuben Garshong, Kaiying Chen, Dac Crossley, Bryan S. Mclean, Gideon Wasserberg, Charles S. Apperson, R. Michael Roe, Loganathan Ponnusamy

**Affiliations:** 1 Department of Entomology and Plant Pathology, Comparative Medicine Institute, North Carolina State University, Raleigh, North Carolina, United States of America; 2 Department of Biology, University of North Carolina at Greensboro, Greensboro, North Carolina, United States of America; 3 Georgia Museum of Natural History, Natural History Building, University of Georgia, Athens, Georgia, United States of America; University of Missouri College of Veterinary Medicine, UNITED STATES OF AMERICA

## Abstract

Chiggers are larval mites that pose a significant health risk globally via the spread of scrub typhus. However, fundamental studies into the bacterial microbiome in North America have never been considered. In this investigation, chiggers were collected in the wild from two locally common rodent host species (i.e., *Sigmodon hispidus* and *Peromyscus leucopus*) in three different ecoregions of North Carolina (NC), United States to investigate the composition of their bacterial communities, including potential pathogens. DNA was extracted from the chiggers, and the V3-V4 regions of the bacterial 16S rRNA gene were sequenced using next-generation sequencing (NGS). Alpha diversity metrics revealed significant differences in bacterial diversity among different collection counties. Beta diversity metrics also revealed that bacterial communities across counties were significantly different, suggesting changes in the microbiome as the environment changed. Specifically, we saw that the two western NC collection counties had similar bacterial composition as did the two eastern collection counties. In addition, we found that the chigger microbiome bacterial diversity and composition differed between rodent host species. The 16S rRNA sequence reads were assigned to 64 phyla, 106 orders, 199 families, and 359 genera. The major bacterial phylum was Actinobacteria. The most abundant species were in the genera *Corynebacterium*, *Propionibacterium*, class ZB2, and *Methylobacterium*. Sequences derived from potential pathogens within the genera *Orientia* and *Rickettsia* were also detected. Our findings provide the first insights into the ecology of chigger microbiomes in the US. Further research is required to determine if the potential pathogens found detected in chiggers are a threat to humans and wildlife.

## Introduction

Chiggers are the vector of scrub typhus, a life-threatening disease in humans. Two decades ago, it was estimated that there were about one million cases of scrub typhus yearly [1]. Since then the number of cases has increased dramatically, being considered endemic in many regions in the Asia-Pacific tsutsugamushi triangle [2–6]. These scrub typhus causing bacteria are closely related to the genus *Rickettsia*; hence, the manifestation of scrub typhus is similar to spotted fever group (SFG) *Rickettsia* illnesses [7]. Until 2010, scrub typhus was thought to only exist in the ‘tsutsugamushi triangle’, which ranges from Pakistan to Russia to Australia [8]. However, knowledge on its distribution is expanding with human cases of scrub typhus recently being reported in Chile [9–11], Peru [12], and the United Arab Emirates [13]. Scrub typhus is caused by the obligate intracellular bacteria *Orientia tsutsugamushi* in the Asia-Pacific Region, *Candidatus* Orientia chuto in the Middle East [13], and *Candidatus* Orientia chiloensis in Chile [14].

Globally, chiggers have been found infected with the causative agents of many bacterial illnesses, including *Orientia* spp. [11, 15–22], *Rickettsia* spp. [18, 23–28], *Borrelia* spp. [29–32], *Bartonella tamiae* [33], *Anaplasma phagocytophilum* [34], and *Coxiella* spp. [21]. Chiggers spread viral pathogens as well, including hantavirus, *Bayou orthohantavirus* and *Hantaan orthohantavirus* [35–38], and the thrombocytopenia syndrome virus, *Dabie bandavirus* [39]. Chiggers were also suspected to be involved in the red meat allergy illness, Alpha-gal Syndrome, which was thought to be exclusively spread by ticks [40]. As previously mentioned, chiggers have also been found infected with SFG *Rickettsia* species [18, 23–26, 28]; however, no Rickettsial human illnesses vectored by chiggers have been reported at this juncture. Recently, free living *Eutrombicula* chiggers collected in North Carolina, USA were found to be infected with *Orientia* spp. [16] but their potential to transmit the pathogen to humans and other animals remains unknown. While it was once thought that scrub typhus did not exist in North America, these recent findings suggest that there is a potential disease risk resulting from chigger bites. Accordingly, the public health threat range of chiggers may be more extensive than initially realized.

Considering that chiggers and ticks both parasitize rodents in the US, it is likely that they share some of the same hosts. With chiggers harboring some of the same SFG species as ticks, it is possible that chiggers could also be responsible for vectoring these pathogenic species. Multiple vital questions arise from these findings, one of which being, are chiggers in the US capable of transmitting tick-borne pathogenic bacteria to humans and other animals? If the former is true, are some human illnesses thought to be tick-borne actually vectored by chiggers?

Scrub typhus is a zoonosis, involving trombiculid mite vectors as well as both wild and urban rodents [15]. Among the different terrestrial vertebrates parasitized by chiggers, rodents stand as competent reservoir hosts to the *Orientia* pathogen. Specifically, *Orientia* spp. have been detected in rodents in France [41], Senegal [41], South Korea [19], Thailand [20], and Kenya [17]. It has been suggested that chiggers may act as both the reservoir and host of pathogens causing scrub typhus [42]. It is widely believed that chiggers feed only once on a host and then drop off to begin molting to the nymphal stages (assuming this feeding was not interrupted) [42–47]. In the nymphal and adult stages, these mites are free-living predators that primarily consume the eggs of other insects [48]. For a pathogen to infect a trombiculid mite, it must be acquired from a vertebrate as larvae, the only parasitic stage of the chigger. If a chigger were to transmit a pathogen to an animal, it would need to be transstadially transferred within the mite and then transovarially transferred to the offspring. Transovarial transmission of *O*. *tsutsugamushi* in chiggers was found to be rare in laboratory studies [49]. However, Chaisiri et al. [48] points out that these rare events are possible especially since chiggers parasitize animals in such large numbers, so transovarial transmission could be less rare in nature.

The development of next-generation sequencing technology has revolutionized our ability to study bacterial communities, including pathogens and symbionts, opening new avenues to answer these questions via characterization of the chigger microbiome. For example, Chaisiri et al. [48] conducted a microbiome analysis of field-collected chiggers from small mammals in Thailand and found complex, highly diverse microbiota and pathogens, including *Orientia* and *Borrelia*. In addition, chiggers also harbor a high abundance of symbiotic and commensal microorganisms that can be obligate or facultative. Another study found a possible mutualistic association between an Amoebophilaceae bacterial species and *O*. *tsutsugamushi* in a lab colony of Thailand chiggers, *Leptotrombidium imphalum* [50]. Currently, there are limited studies that have investigated the chigger microbiome and even less that have analyzed microbiome composition differences in host-associated chiggers. Ours is the first study to investigate the microbiome of North American chiggers, and it adds the novel component of a comparison of the microbiome of chiggers from different ecoregions, differing chigger species, and collected from two different rodent species. Chiggers are highly prevalent in the US, being reported in every state except Hawaii [51]. Despite the public health importance of trombiculid mites worldwide, we know nothing about the diversity and composition of their microbial communities in the US. Given this, our specific study goals were (i) to determine the species composition and diversity of the rodent associated chigger’s bacterial microbiome, (ii) to determine whether chigger microbiomes varied in composition between different rodent host species, and (iii) to ascertain if there were differences in the microbiome composition of chiggers from the three major ecological regions of North Carolina: mountain, piedmont, and coastal plains.

## Materials and methods

### Site description of rodent trapping

**Fig 1:**
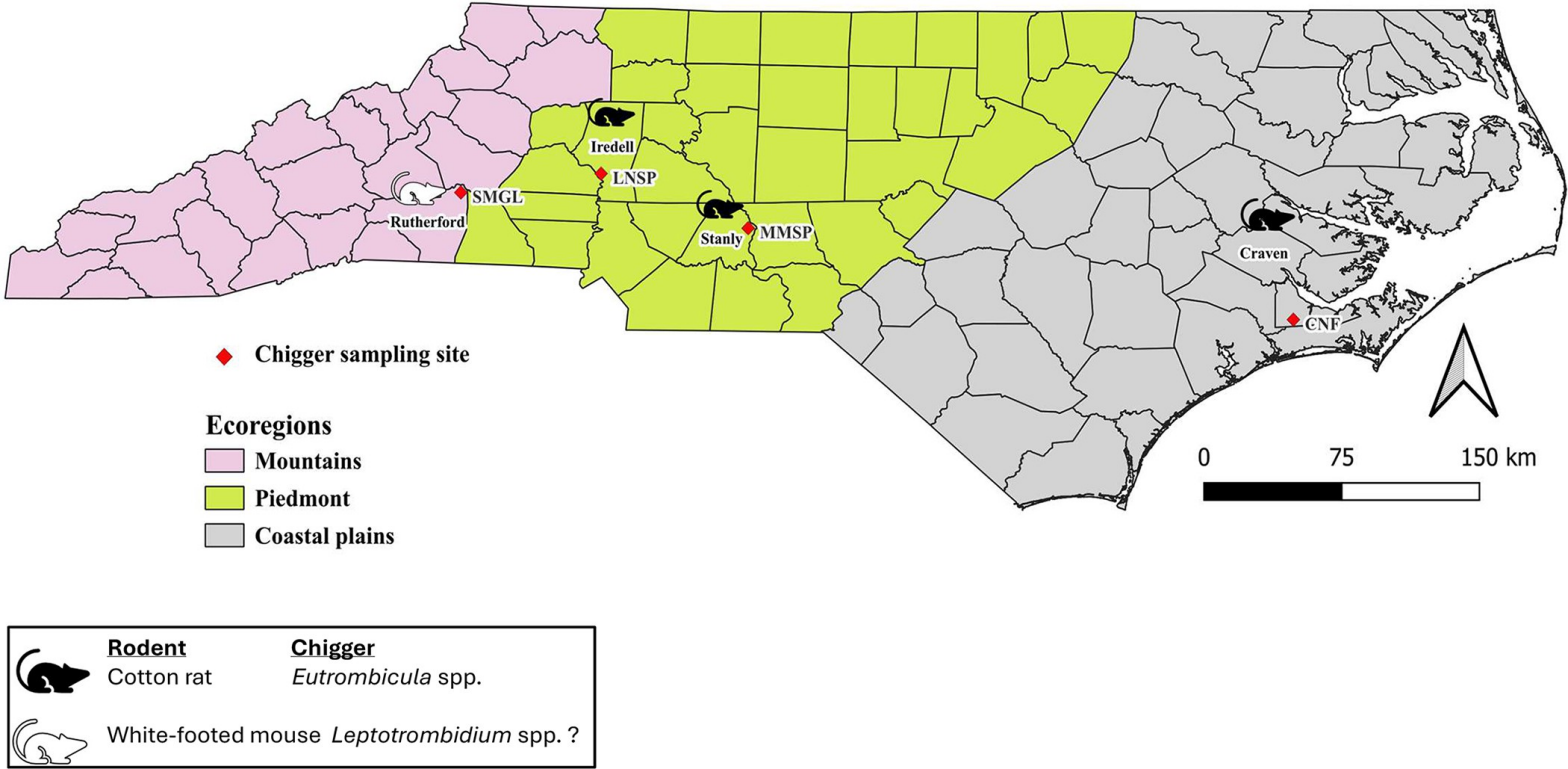
Map of collection sites where rodent-associated chiggers were sampled. The three ecoregions of North Carolina are indicated in different colors. The red dots represent the geographic location where chiggers were collected off live rodents. The acronyms in uppercase letters below the red dots represent the different chigger collection sites: SMGL—South Mountains Game Lands, LNSP—Lake Norman State Park, MMSP—Morrow Mountain State Park, and CNF—Croatan National Forest. The county names are indicated in black text cases above the red diamond shape. The hispid cotton rat is represented by the black rodent symbol and the white-footed mouse by the white rodent symbol. The genus of chigger collected is indicated in the bottom key next to the rodent type. The chiggers collected from the white-footed mouse were morphologically identified to be *Leptotrombidium* spp. This map was created using a free, open-source quantum geographic information system (QGIS, version 3.34.6). The North Carolina shapefile was modified from the USA shapefile from gadm.org (GADM data, version 4.1).

Four field sites were sampled within four counties of North Carolina, USA. The sites were: South Mountains Game Lands (SMGL) in Rutherford County, Lake Norman State Park (LNSP) in Iredell County, Morrow Mountain State Park (MMSP) in Stanly County, and Croatan National Forest (CNF) in Craven County (Fig 1). SMGL is in western NC within the mountain ecoregion. Its landscape is comprised mainly of shortleaf pine (*Pinus echinata*), oak (*Quercus* spp.) heath, oak (*Quercus* spp.), hickory (*Carya* spp.) hardwood forests and open meadows that contain ferns (Pteridophyta), herbs, grasses (Poaceae), and young regenerative trees. LNSP includes a mix of hardwood and pine (*Pinus* spp.) forest with hickories (*Carya* spp.), sweetgum (*Liquidambar* spp.), red maple (*Acer rubrum)*, dogwood (*Cornus* spp.), and oaks (*Quercus* spp.) constituting the hardwood. Close to the lake’s edge are communities of alder (*Alnus* spp.), willow thickets (*Salix* spp.), marshes of grasses, rushes, and sedges (*Carex* spp.). In MMSP, mesic dry oak (*Quercus* spp.), hickory (*Carya* spp.) forest and other hardwoods such as red maple (*Acer rubrum*) and sourwood (*Oxydendrum arboreum*) are interspersed with pine (*Pinus* spp.), herbs, and sedges (Cyperaceae). LNSP and MMSP lie within the Piedmont ecoregion (Fig 1). CNF is in the Coastal Plains ecoregion where pine (*Pinus* spp.) forests, old growth beech (*Beech* spp.), oak (*Quercus* spp.) forests, saltwater estuaries, and evergreen-shrub bogs (pocosins) are present. It also has bottomland oaks (*Quercus* spp.) and cypress trees (*Cupressus* spp.), among others. All the sites are accessible to the public for various recreational activities such as hiking, boating, fishing, hunting, birding, camping, and picnicking.

### Rodent trapping and chigger collection

Rodents were live trapped using aluminum Sherman live animal traps (HB Sherman Traps Inc., Tallahassee, FL, USA) and Longworth traps (Anglian Lepidopterist Supplies, Norfolk, England) during the summers of 2020 (only for SMGL) and 2022 (for the remaining three sites) (Fig 1). Two different trap types at each site were used as it has been noted that the use of the two different trap types simultaneously helps to improve small mammal capture efficiency [52]. However, both rodent types were found in both sites. Peanut butter (Great Value Creamy Peanut Butter, Walmart Stores Inc., Bentonville, AR, USA) mixed with dried oats (Great Value Old Fashioned Oats, Walmart Stores Inc., Bentonville, AR, USA) was used as bait [23]. Traps were set during sunset and inspected at sunrise daily for three consecutive days. Each trap was closed after morning inspections and reopened at sunset. There were at least 80 traps per night at each site. Each rodent was screened for chiggers. The caught rodents were then systematically examined for attached chiggers by observing the less furry parts of the rodent’s body, particularly in and around the ears, around the feet, abdomen area, and around the genitals as blood-feeding ectoparasites are not easily dislodged in these areas [53]. For the furry parts of the rodent, a louse comb (Oster Animal, Sunbeam Products, Inc., FL, USA) was used to lift the pelage to detect attached chiggers on those parts of the rodent’s body. Attached chiggers appeared as clusters of orange-red spots [54]. Rodent-associated chiggers were collected off the animal’s skin using sterile forceps or a louse comb. The collected chiggers were immediately stored in 1.5-mL Eppendorf tubes or glass vials containing 95% ethanol (Fisher Scientific, Hampton, NH, USA). Chiggers that were collected from the same animal were put in the same tube and stored at −20°C in the laboratory. Each rodent captured was sexed and identified using the Peterson Field Guide to Mammals of North America, North of Mexico [55]. Rodents that were recaptured within the three consecutive trapping days (identified by ear punch) were re-examined for chiggers and released at the same point of capture.

### Ethics statement

The collection of rodents and handling techniques followed the guidelines and protocols of the American Society of Mammalogists [56] and the University of North Carolina at Greensboro Institutional Animal Care Use Committee (IACUC 21–006 and 20–008 for Wasserberg and McLean labs, respectively). Permits to trap rodents and collect associated ectoparasites in the different sites were obtained from the North Carolina Wildlife Resources Commission and the Forest Service of the US Department of Agriculture.

### DNA extraction of chiggers

DNA from some of the chiggers (C1-16 and C21-49) was extracted in a previous study [23]. An additional, 30 chiggers (C50 to C79) were surface sterilized following the methods described in Ponnusamy et al. [50]. Total genomic DNA was extracted from each chigger using the QIAGEN DNeasy Blood & Tissue Kit, following the manufacturers’ instructions (QIAGEN, Valencia, CA, USA). After surface sterilization, each chigger was transferred to a separate microcentrifuge tube with about 10 sterilized 3-mm glass beads (Cat. 11-312A, Fisher Scientific), and appropriate volumes of ATL buffer, and Proteinase K solution as stated in the DNA extraction kit protocol. Following this, each sample was homogenized using a FastPrep FP120 cell homogenizer (Thermo Electron Corporation, Waltham, MA, USA), and then incubated at 56°C for 1 h. Next, an AL buffer was added to each sample, mixed, and incubated at 56°C for 1 h. DNA was then purified and eluted using 30 μL of nuclease-free water and stored at −20°C until use.

### Identification of chigger samples

For morphological identification, 10–15% of the numbers of chiggers from each rodent were identified to genus and species, using previously described identification keys [57–59]. Additionally, 5 chiggers from each county were chosen for molecular identification through previously described 18s ribosomal RNA gene primers and PCR [60]. Amplicons were then Sanger sequenced at Eton Bioscience, Inc. (Research Triangle Park, NC, USA). The sequences obtained were compared to existing genes within GenBank to identify the chiggers.

### 16S rRNA amplification, Illumina library construction, and sequencing

Bacterial 16S rRNA sequencing libraries were constructed according to Illumina’s 16S rRNA metagenomics sequencing library preparation protocol (Illumina, San Diego, CA, USA). Briefly, the initial round of PCR was carried out using 341F/806R universal primers with Illumina adapter overhang sequences [50, 61]. After the PCR amplification, PCR products were purified, and additional PCR amplification conducted using the Nextera XT Index Kit (Illumina, USA). The constructed 16S rRNA libraries were quantified with Quant-iT PicoGreen (Molecular Probes, OR, USA), and the libraries were normalized and pooled prior to sequencing. Samples were then paired-end sequenced (2 x 300 bp) on an Illumina MiSeq sequencer at the Microbiome Core Facility in The School of Medicine, University of North Carolina at Chapel Hill (Chapel Hill, NC, USA).

#### Microbiome sequence accession numbers

Raw sequences were submitted to the NCBI Sequence Read Archive (SRA) with the accession number under the BioProject SUB14351237. Data sequences are available at SRA data: PRJNA1106524.

### Bioinformatics data processing

Chigger microbiome analysis was conducted using Quantitative Insights into Microbial Ecology 2 (QIIME2) and its associated plugins [62]. The FASTQ files were demultiplexed and quality-filtered (Q > 20). Reads were then denoised, filtered, paired-ends merged, chimera removed using the Divisive Amplicon Denoising Algorithm 2 (DADA2), trimmed (—p-trim-left-f 17,—p-trim-left-r 21) and then truncated (trunc-len-f 275 and trunc-len-r 215) to remove low quality sequences [63]. Using the *Align-to-tree-mafft-fasttree* pipeline, sequences were aligned, and a phylogenetic tree constructed using the q2-phylogeny plugin of QIIME 2 [62]. Next, taxonomy classification was performed using a trained Naive Bayes classifier from Greengenes 13_8 with 99% sequence similarity to the OTU (observational taxonomic unit) data set [64].

The q2-diversity plugin pipeline was used to evaluate the alpha and beta diversity of these samples with a sampling depth of 1000. The alpha diversity measures the within sample diversity by the Shannon’s diversity index, Faith’s Phylogenetic Diversity and the Observed Features measures. Shannon’s diversity index measures the richness and evenness of the samples [65]. Faith’s Phylogenetic Diversity calculates the phylogenetic branch distance lengths [66]. Observed Features measures the richness of unique sequence variants and estimates species richness [63]. The beta diversity was measured using the weighted and unweighted UniFrac distance measure, and then the EMPeror [67] plugin was used to visualize the principal coordinate analysis (PCoA) figures. Multiple variables were investigated during these analyses including the collection county, host type, ecoregion, and individual rodent. An ANCOM analysis [68, 69] was conducted using QIIME2 to identify the significant differentially abundant bacterial taxa at the genus level among collection counties and hosts. We used a Venn Diagram to illustrate the overlapping genera between the groups. Venn diagrams were built for visualizing unique and common ASVs (amplicon sequence variants) among all groups using the “InteractiVenn” online program [70].

### Phylogenetic analyses of *Rickettsia* and *Orientia* sequences

To further confirm and/or improve the phylogenetic relationships, we extracted 11 *Rickettsia* and three *Orientia* ASVs of 16S rRNA sequences from the microbiome sequences data. Two separate phylogenetic analyses were performed with other closely related *Rickettsia* and *Orientia* sequences acquired from the NCBI database (accessed on December 5, 2023). Using the ClustalW program [71], multiple alignments were conducted for both genera. The phylogenetic trees were constructed using the maximum likelihood (ML) analysis with the Kimura two-parameter model [72] in MEGA 11 software [73]. Bootstrapping at 1000 re-sampling iterations were calculated for each tree. Additionally, a pairwise distance analysis was performed using MEGA 11 software [73] to obtain all sequence similarity values.

## Results

### Chigger collections and identification

A total of 75 chiggers used in this study were collected from four hispid cotton rats, *Sigmodon hispidus*, and four white-footed mice, *Peromyscus leucopus*. The white-footed mice were caught in South Mountain Gameland (SMGL; Rutherford County) while the hispid cotton rats were all from the remaining three locations (Fig 1). No chiggers were found on any recaptured rodents. Based on the morphological identification, chiggers collected in Iredell, Stanly, and Craven Counties (*n* = 47) were in the genus *Eutrombicula* (Table 1). Chiggers collected within Rutherford County (*n* = 28) were identified in the genus *Leptotrombidium* (Table 1). The sub-set of chiggers collected from cotton rats from three other collections were morphologically identified as *Eutrombicula* spp. Of the subset of 20 chiggers identified using the 18S rRNA molecular marker, 60% (*n* = 12) were high quality sequences and 40% (*n* = 8) were ambiguous. The high-quality sequences revealed that the chiggers from Iredell, Stanly, and Craven Counties had 96.91–100% sequence similarity to the genus *Eutrombicula* spp. which matched our morphological identification. The chiggers from Rutherford County had 93.85–94.23% sequence similarity to the genus *Pseudoschoengastia* and 93.08–93.46% sequence similarity to the genus *Eutrombicula* but were morphologically identified to be within the genus *Leptotrombidium*. These results are summarized in Table 1.

**Table 1:** Summary of all chigger collection results obtained in this study. This table includes the collection county chiggers were collected in, the number of chiggers collected, the morphological and molecular ID of chiggers to genus, the host type the chigger was collected from, the ecoregion the chigger was collected in, and the number of individual rodents the chiggers were collected from. The morphological ID of chiggers to genus was completed using previously described identification keys and molecular ID of chiggers was completed using 18s ribosomal RNA gene primers and PCR.

Counties	Number of Chiggers Collected	Morphological ID of Chiggers to Genus	Molecular ID of Chiggers to Genus	Host Type	Ecoregion	Number of Individual Rodents (Rodent ID)
Rutherford	28	*Leptotrombidium* spp.	Unknown	White-footed mouse	Mountain	4 (R2-R5)
Iredell	17	*Eutrombicula* spp.	*Eutrombicula* spp.	Hispid Cotton rat	Piedmont	1 (R1)
Stanly	20	*Eutrombicula* spp.	*Eutrombicula* spp.	Hispid Cotton rat	Piedmont	2 (R6 & R7)
Craven	10	*Eutrombicula* spp.	*Eutrombicula* spp.	Hispid Cotton rat	Coastal Plains	1 (R8)

### Bacterial 16S rRNA gene amplicon sequencing results

A total of 4,829,540 sequencing reads were produced by Illumina sequencing from 75 chigger samples. The taxa Streptophyta made up 11.5% of the initial chigger microbiome, and were excluded from further analysis. After the removal of Streptophyta and quality filtering with the DADA2 algorithm, 1,469,342 reads remained with an average of 19,591.23 reads per chigger sample. The total bacterial 16S rRNA reads were assigned to 64 phyla, 106 orders, 199 families, 359 genera and 444 species-level ASVs (amplicon sequence variants).

### Alpha diversity

**Fig 2:**
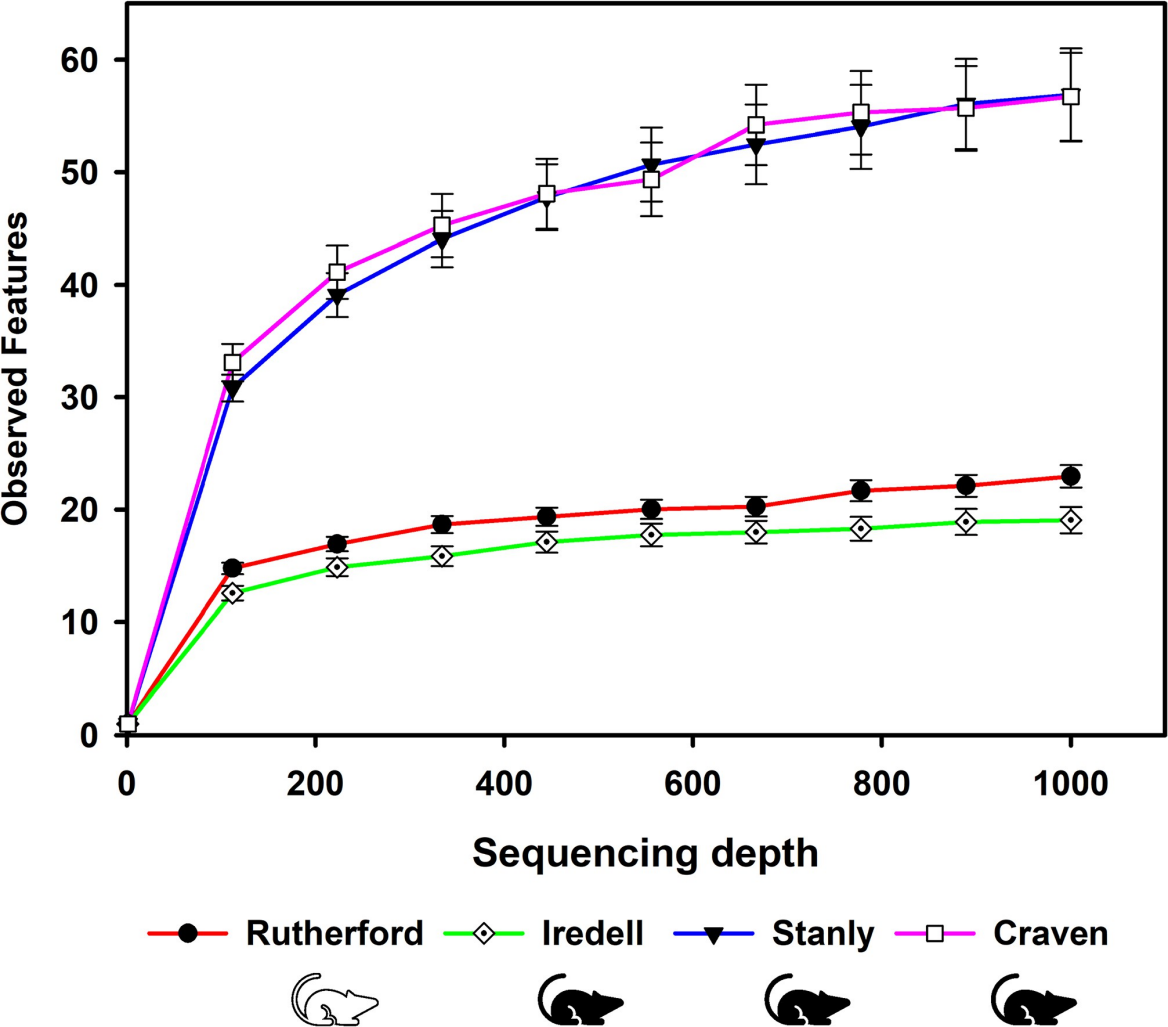
Rarefaction curves of the mean number of observed features (ASVs) (amplicon sequence variants) (y-axis) in North Carolina, USA, where chiggers were collected off rodents. Error bars represent the standard error of the mean. The hispid cotton rat is represented by the black rodent symbol and the white-footed mouse by the white rodent symbol.

**Fig 3:**
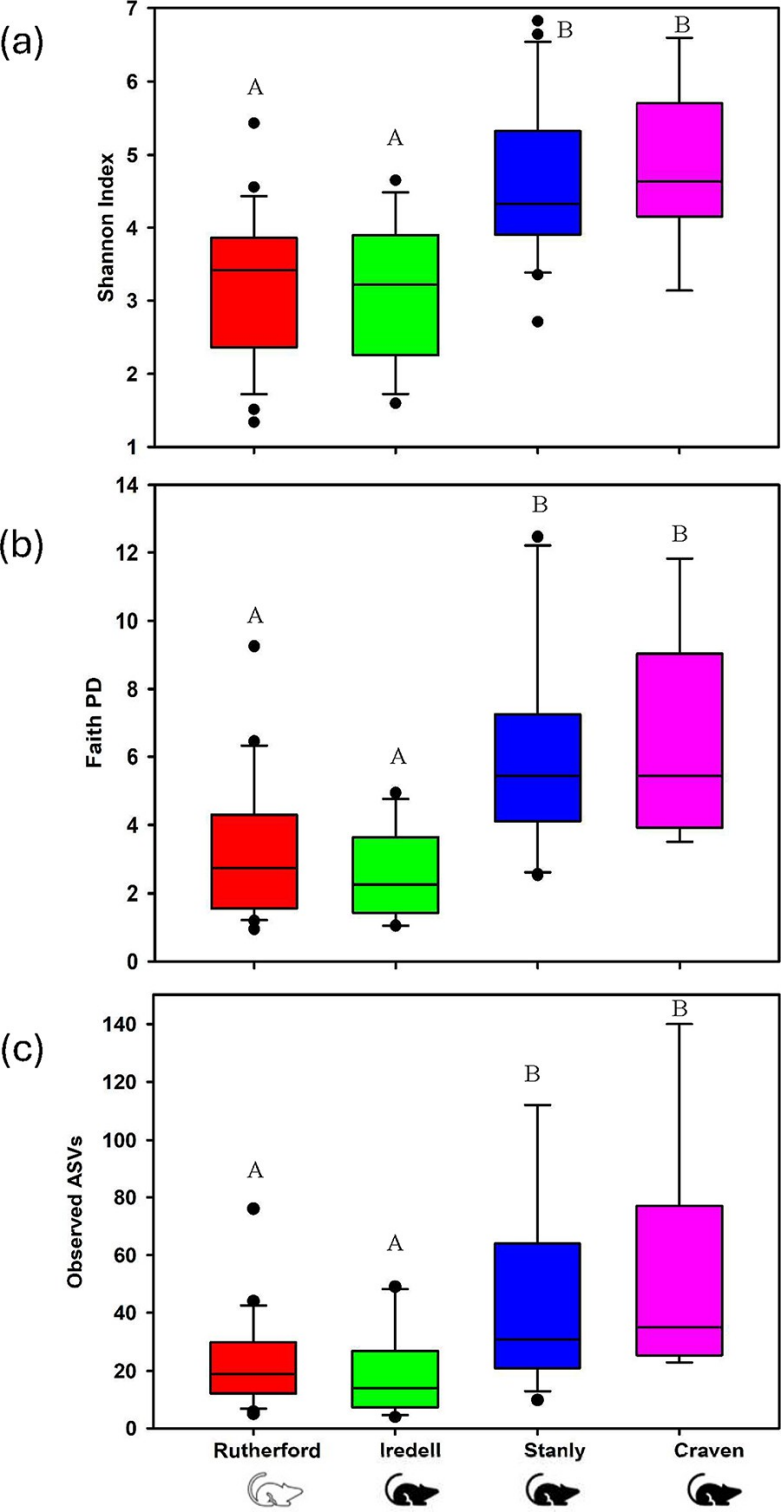
Alpha diversity measures of the microbiome of chiggers collected from rodents in Rutherford, Iredell, Stanly, and Craven counties, North Carolina, USA. (a) Shannon diversity, (b) Faiths phylogenetic diversity, and (c) Observed (ASVs). Letters A and B are shown above each box to show significant differences. The horizontal lines on the bottom of box plots denoting the lower interquartile value, the middle line is the median, and the top line is represented by the upper interquartile value. Filled circles outside of the line are outliers. The hispid cotton rat is represented by the black rodent symbol and the white-footed mouse by the white rodent symbol.

The rarefaction curves of the observed ASVs showed that a sequencing depth of 1000 was sufficient to retrieve most of the bacterial taxa present in the samples (Fig 2). Nine samples with lower sequencing depth (below 1000 sequencing reads) were removed from the diversity analysis. The bacterial alpha diversity patterns of chiggers were explored using observed features, Shannon’s diversity index, and Faith’s phylogenetic diversity index. In the Shannon index analysis conducted at the level of collection counties, there were differences among all groups (Kruskal-Wallis, H = 25.04; *p* < 0.001; Fig 3A). The Faiths phylogenetic diversity (PD) index revealed that among the county groups, there were also statistically significant differences in diversity (Kruskal-Wallis, H = 24.4; *p* < 0.001; Fig 3B). These two metrics differ in that the Shannon index is sensitive to species richness as well as evenness, while the Faiths PD is a measure of richness that is made up of the sum of the phylogenetic tree branch lengths. Like the two other alpha diversity measures, the number of observed ASVs were significantly different among all county groups and showed high diversity (Kruskal-Wallis, *H* = 16.94; *p* < 0.001; Fig 3C). For all three of these analyses, the pairwise comparisons showed that the microbiome of chiggers from Craven and Stanly counties did not differ from each other (Kruskal-Wallis pair wise, *p* > 0.05; Fig 3) and this was also true for the pairwise analysis of Iredell and Rutherford counties (Kruskal-Wallis pair wise, *p* > 0.05; Fig 3). However, all other county comparisons (Craven-Rutherford, Craven-Iredell, Stanly-Rutherford, and Stanly-Iredell) showed to be statistically significantly different from each other (Kruskal-Wallis pair wise, p < 0.05) for the Shannon index, Faiths PD, and the observed features analysis.

When comparing microbiome diversity between the two rodent host species, the alpha diversity proved to be higher in chiggers from the hispid cotton rat than those from the white-footed mouse (Shannon index; *p* < 0.05; S1 Fig). Similarly, this difference was also found to be statistically significant when using the Faiths PD or the observed features index (*p* < 0.05; S1 Fig). Notably, the sample size for the cotton rat was much larger (*n* = 42) than for the white-footed mouse (*n* = 24). Two different chigger taxa were identified in this study, one only on the hispid cotton rat and one only on the white footed mouse (Table 1). Significant differences were observed when comparing the microbiome of chiggers between eight different individual rodents (S2 Fig).

### Beta diversity

**Fig 4:**
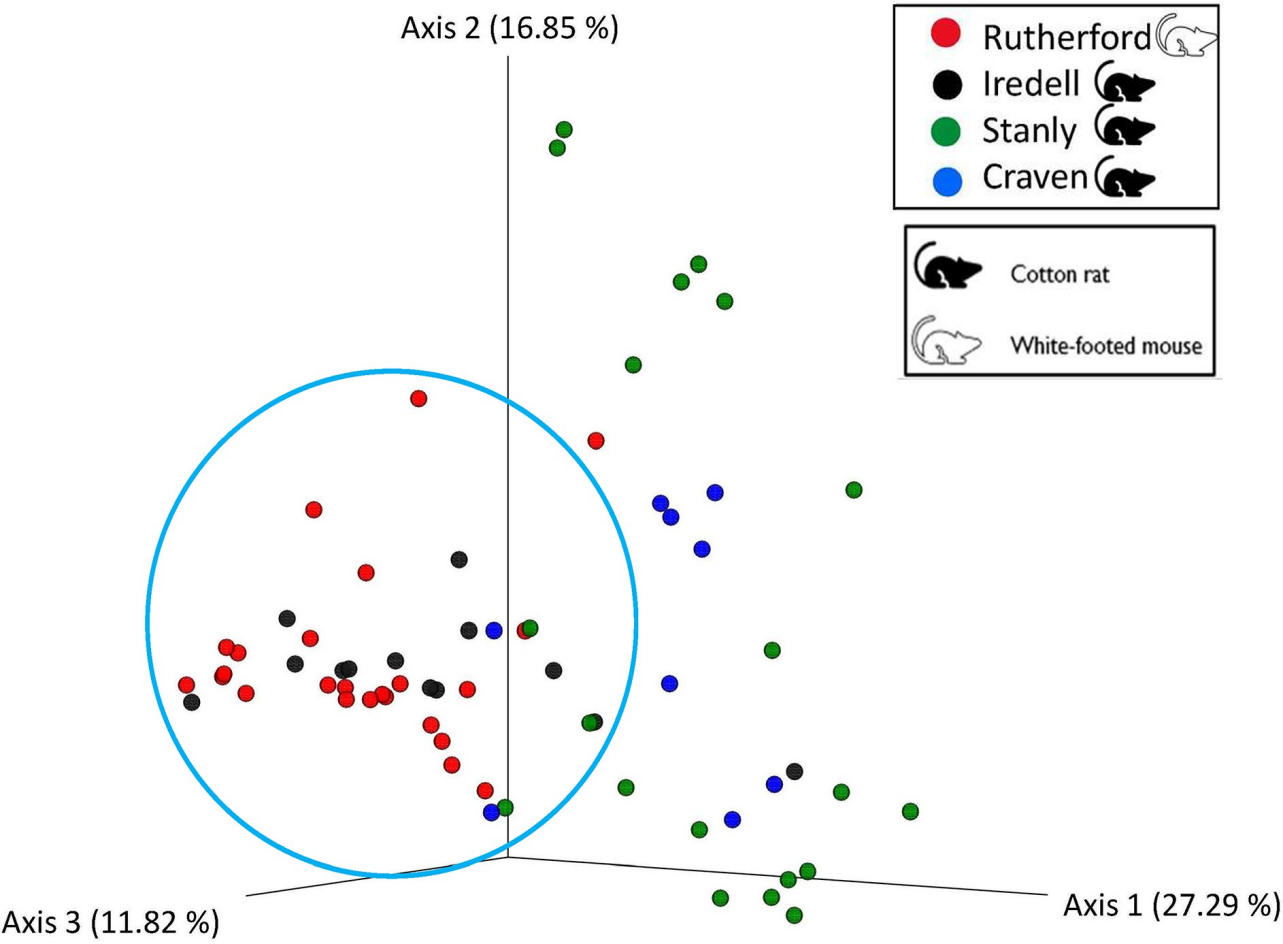
Principal coordinate analysis (PCoA) of bacterial composition in chiggers collected from rodents in Rutherford, Iredell, Stanly, and Craven counties, North Carolina, USA. The analysis was based on the weighted UniFrac metric and visualized using Emperor. The circle points out clustering among the collection counties. The key shows the color used to represent each county and the rodent type that was collected in that county.

**Fig 5:**
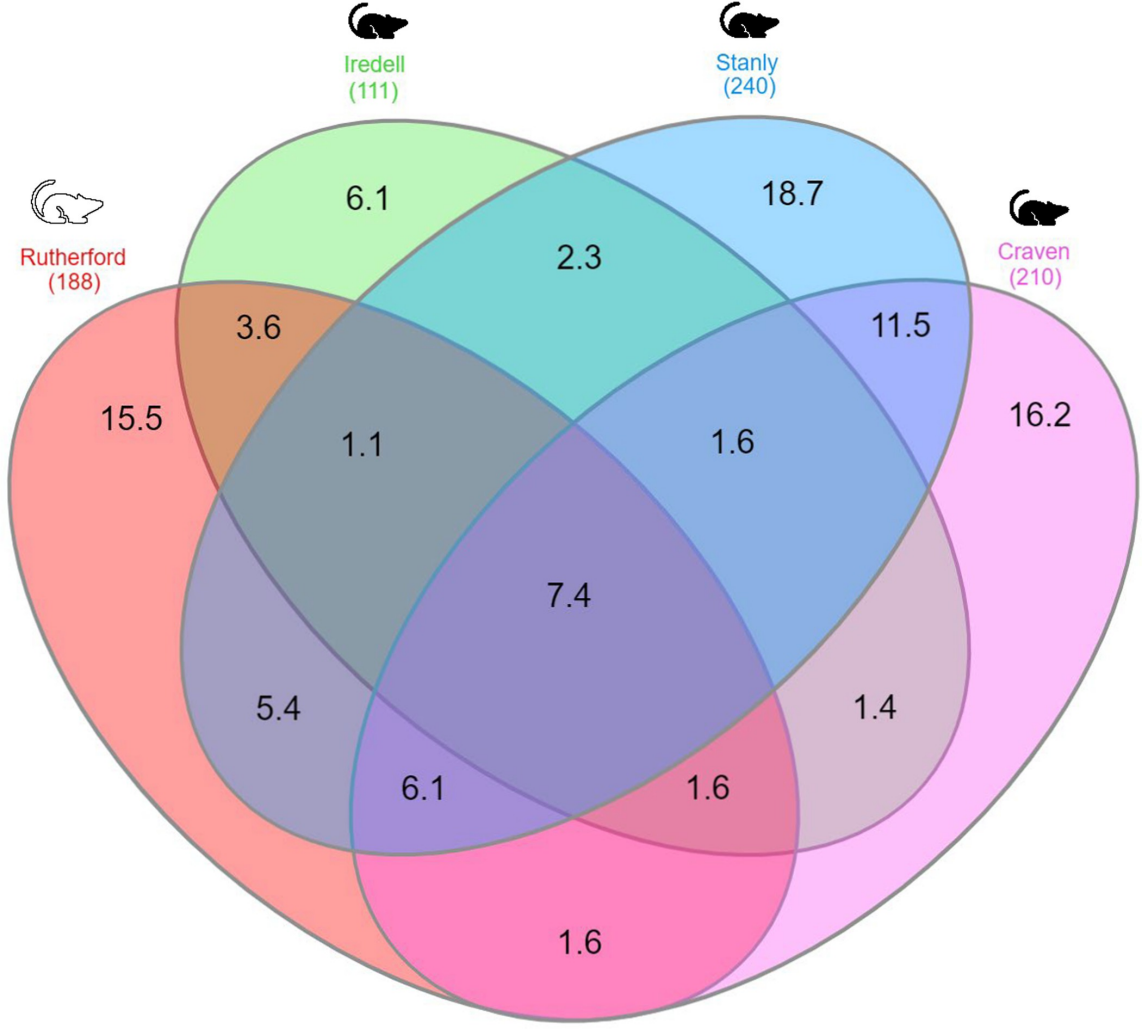
Comparison of unique and shared bacterial taxa found in chiggers collected in different counties in North Carolina, USA. Venn diagram shows the percentages of the common and different predicted bacterial species level taxa (level 7) found in the microbiome of chiggers. The hispid cotton rat is represented by the black rodent symbol and the white-footed mouse by the white rodent symbol.

To estimate beta diversity, for the four counties, we measured their weighted UniFrac distance. This analysis revealed some clustering between Rutherford and Iredell Counties, and there was significant differences seen among the county groups in the PERMANOVA analysis (Fig 4; PERMANOVA, *p* = 0.001). Significant differences were assessed through the pairwise PERMANOVA analysis and results are summarized in Table 2. Two additional principal coordinate analyses (PCoA) showed the bacterial composition in chiggers amongst the two different host species (hispid cotton rat and white-footed mouse) (S3 Fig) and the eight individual rodents from which chiggers were collected (labeled R1-R8) (S4 Fig). Distinct clusters can be seen between the two host species in S3 Fig (PERMANOVA, *p* = 0.001). Additionally, no distinct clusters can be seen in the PCoA displaying all of the individual rodents from which chiggers were collected (S4 Fig) (PERMANOVA, *p* = 0.001). The bacterial quantity for each taxon was visualized by county using a Venn-diagram showing the unique and shared taxa between the four collection counties (Fig 5). Of the 444 species-level ASVs, only 36 (7.4%) were shared by all the collection counties. There were 27 ASVs (6.1%) unique to Iredell, whereas 69 (15.5%), 83 (18.7%), and 72 (16.2%) were unique to Rutherford, Stanly, and Craven respectively (Fig 5). When comparing the species-level ASVs organized by the two host species, there were 119 (26.8%) shared by both hosts, 256 (57.7%) unique to the hispid cotton rat, and 69 (15.5%) unique to the white-footed mouse (S5 Fig).

**Table 2:** Pairwise PERMANOVA analysis results for each chigger collected from rodents in Rutherford, Iredell, Stanly, and Craven counties, North Carolina, USA based on the weighted UniFrac. “County” column compares one chigger collection county to multiple counties in the column “counties”.

County	Counties compared	Sample Size	*p*-value
Craven	Iredell	22	0.0015
	Rutherford	33	0.0015
	Stanly	29	0.0156
Iredell	Rutherford	37	0.0440
	Stanly	33	0.0015
Rutherford	Stanly	44	0.0015

### Taxonomic analysis of 16S rRNA gene sequencing data

**Fig 6:**
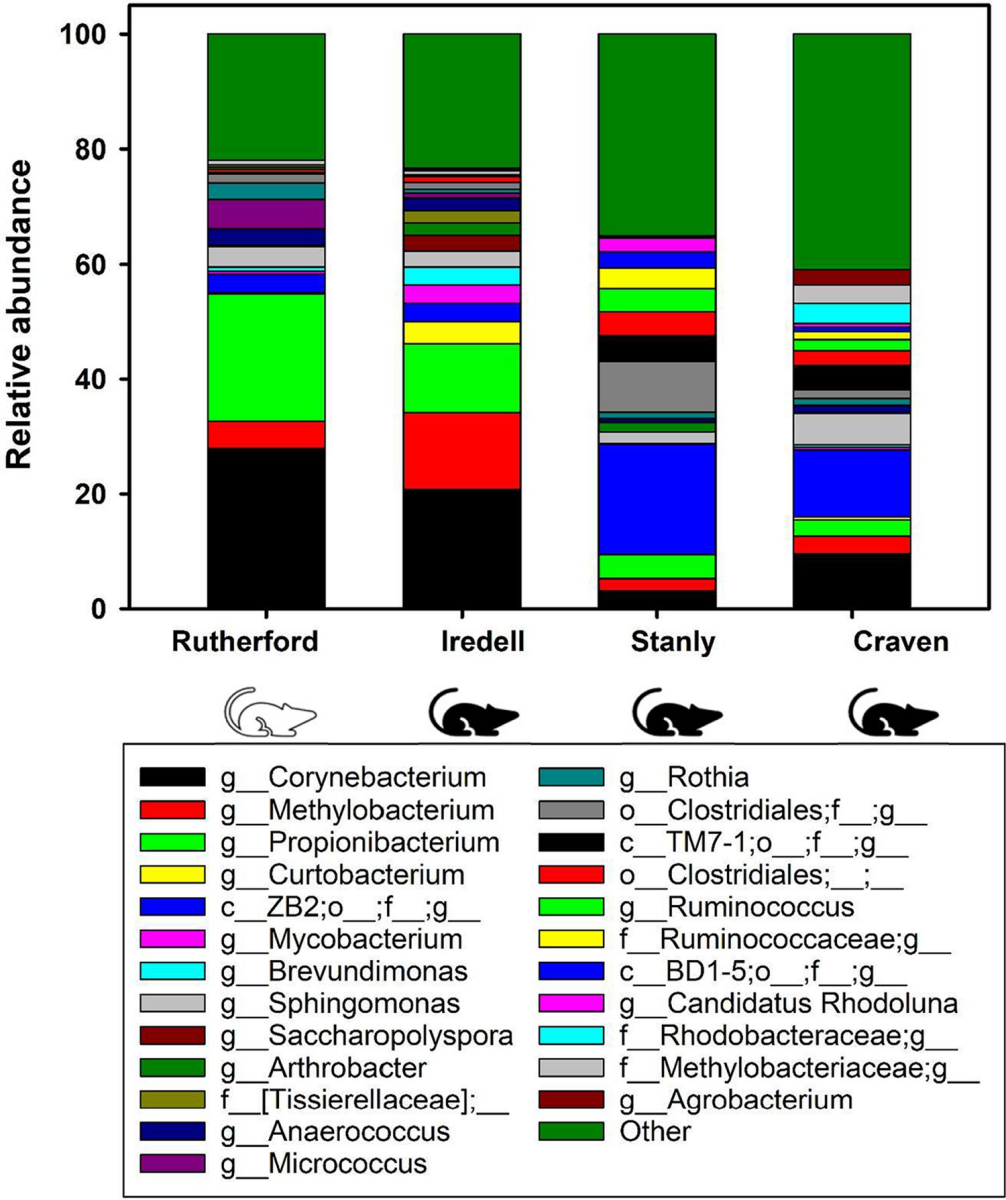
Relative abundances of major bacterial taxa at the genus level compared for chiggers collected from rodents in Rutherford, Iredell, Stanly, and Craven counties, North Carolina, USA. The ‘Other’ category represents all taxa with relative abundance below 2%. Some taxa were only able to be identified up to the genus level. In the legend, the “f_” represents family; “p_” represents phylum, and “c_” represents class. The hispid cotton rat is represented by the black rodent symbol and the white-footed mouse by the white rodent symbol.

**Fig 7:**
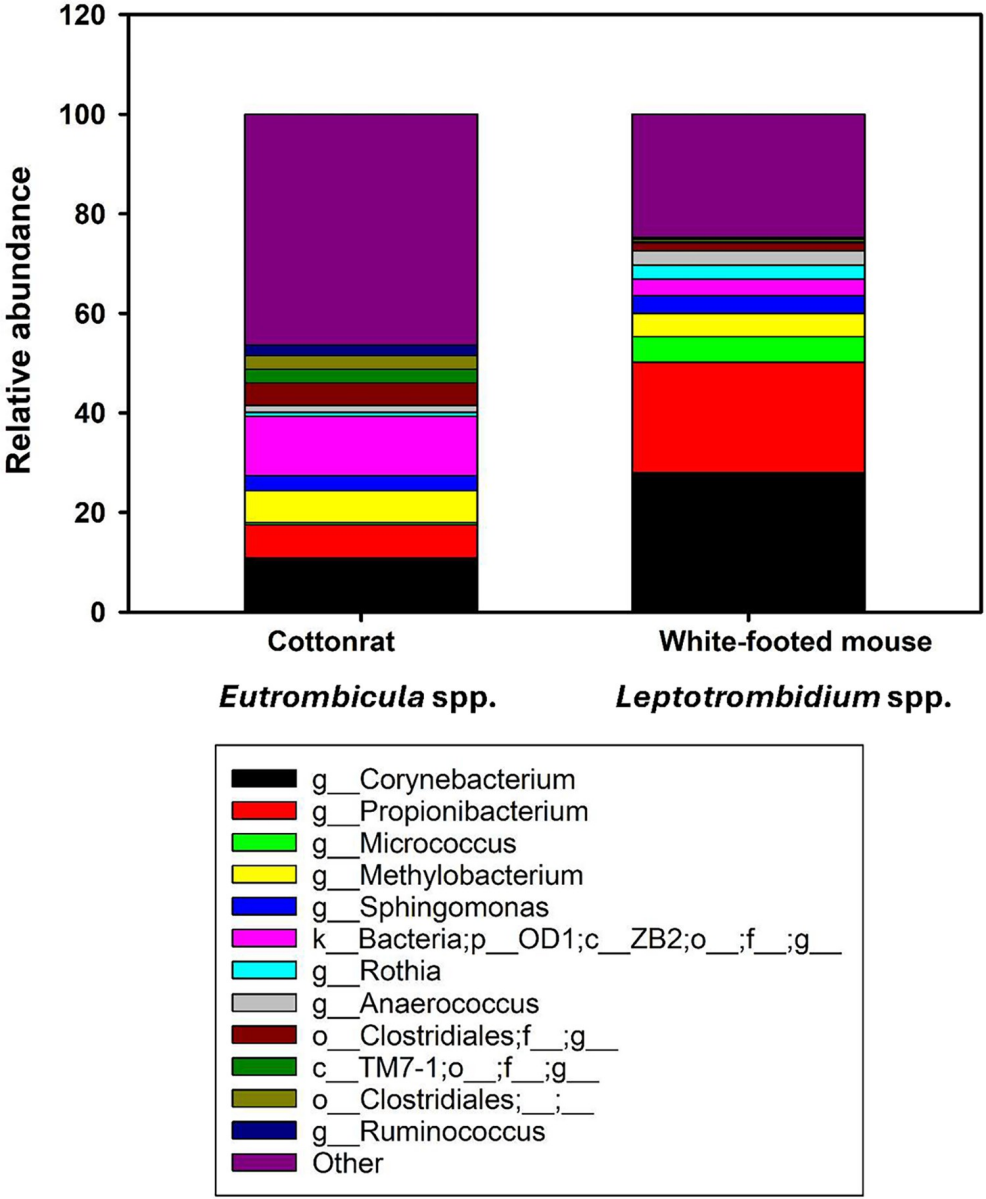
Relative abundances of major bacterial taxa at the genus level compared among the two host types (hispid cotton rat and white-footed mouse). The ‘Other’ category represents all taxa with relative abundance below 2%. Each stacked bar graph represents the mean sample composition for both host types. Some taxa were only able to be identified up to the genus level. “f_” represents family; “p_” represents phylum, and “c_” represents class.

The four most commonly identified genera based on total relative abundance in the chiggers were *Corynebacterium* (17.2%), *Propionibacterium* (12.5%), the class ZB2 (8.6%), and *Methylobacterium* (5.8%). *Corynebacterium*, *Propionibacterium*, and *Methylobacterium* had higher relative abundance in the counties of Iredell (20.8%, 12.1%, and 13.4%, respectively) and Rutherford (27.9%, 22.2%, and 4.7% respectively) whereas the class ZB2 was more abundant in Stanly (19.3%) and Craven Counties (11.7%). Fig 6 depicts a comparison of the bacterial composition and relative abundance among the four collection counties. The bacterial relative abundance differences in chiggers between host species can be seen in Fig 7. The chiggers collected from the white-footed mouse had a higher abundance of *Propionibacterium* and *Corynebacterium* than chiggers collected from the hispid cotton rat (Fig 7). Overall, the chiggers collected from the hispid cotton rat had greater bacterial diversity with a higher number of bacterial taxa identified in these chiggers (Fig 7 and S5 Fig). However, a larger sample size was collected from the hispid cotton rat (*n* = 4 rodents, n = 47 chiggers) compared to the white-footed mouse (*n* = 4 rodents, n = 28 chiggers). Additionally, each rodent species had a different species of chigger parasitizing it.

The potentially pathogenic genera, *Rickettsia* spp. made up 0.66% of the chigger microbiome among all chigger samples, infecting 11 (16.7%) individual chiggers representing all four collection counties and collected from both host types. *Orientia* spp. made up 0.38% of the chigger microbiome, infecting 4 (6.1%) individual chiggers from Stanly and Craven County, only from cotton rats. The genus *Wolbachia*, an endosymbiotic bacterium, was present in 4 (6.1%) chiggers collected from Stanly and Craven County on hispid cotton rats, making up an average of 0.096% of the overall chigger microbiome.

### Differential abundance

The ANCOM test visualized by the volcano plot in S6 Fig, showed that seven ASVs differed among different counties. The ANCOM test showed that *Corynebacterium* was highly abundant among the chiggers from all counties, the highest being in Rutherford County (S6 Fig). The microbiome of the chiggers collected from Rutherford County also had the highest sequence reads for *Propionibacterium* compared to the other counties. In Iredell County and Rutherford County, one or fewer sequences assigned to *Candidatus* Rhodoluna were observed at all percentiles. This finding was also seen at the 50^th^ and 75^th^ percentiles in Craven County. One or fewer sequences were identified in the family Rhodobacteraceae in all percentiles within the counties Iredell and Rutherford as well as most of Stanly County, excluding the 100^th^ percentile. There was one or fewer sequences identified in the class BD1-5 in Iredell and Rutherford Counties.

### Phylogenetic analyses of *Orientia* spp. and *Rickettsia* spp.

**Fig 8:**
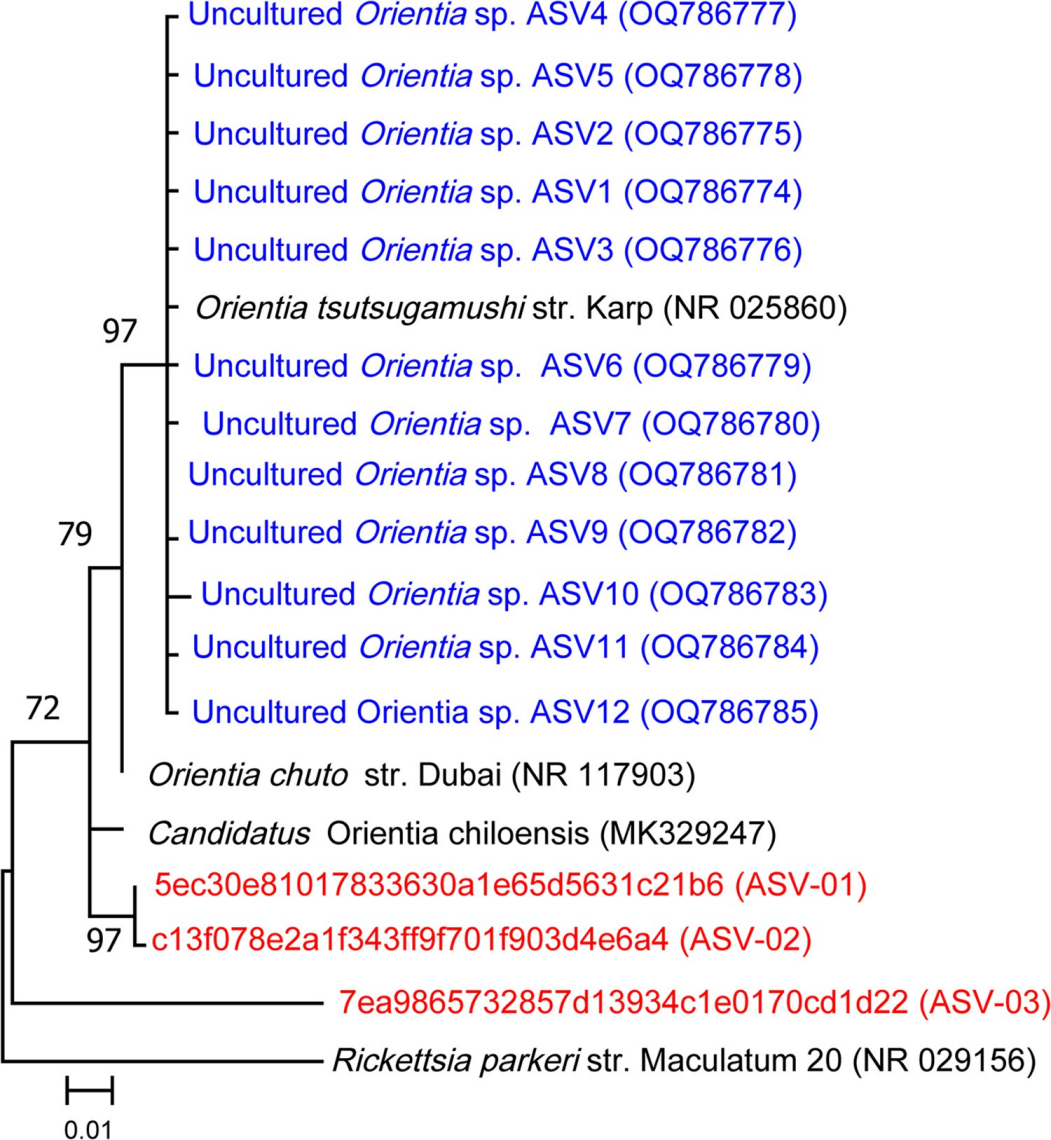
Maximum likelihood phylogenetic analysis of *Orientia* 16S rRNA gene amplicon sequence variants (ASVs). ASVs identified in this study are shown in red, sequences from study on free living chiggers in blue (and *), and closest related species black. Numbers at nodes represent bootstrap values (%), which were obtained from 1000 replicates; values <50 % are not shown. Bar, 0.01 substitutions per nucleotide position. Each ASV has an identification label in parentheses next to it. *Rickettsia parkeri* strain (NR029156) was included as an outgroup.

Three total ASVs were examined in the genus *Orientia*, and a phylogenetic tree was created, including these three ASVs, other recently identified *Orientia* species found in free-living chiggers in North Carolina, and the closest related species (Fig 8). The phylogeny suggests that 2 of the ASVs clustered most closely to *O*. *chiloeensis*. A pairwise distance analysis revealed that these ASVs (ASV-O1 and ASV-O2) were 98.23% and 97.97% similar to *O*. *chiloeensis*. ASV-O3 was 90.71% similar to *O*. *chiloeensis*. The pairwise distance analysis also showed that ASV-O1 and ASV-O3 are 91.56% similar to each other. ASV-O2 was over 99.75% similar to sequence ASV-O1 and 91.28% similar to ASV-O3.

**Fig 9:**
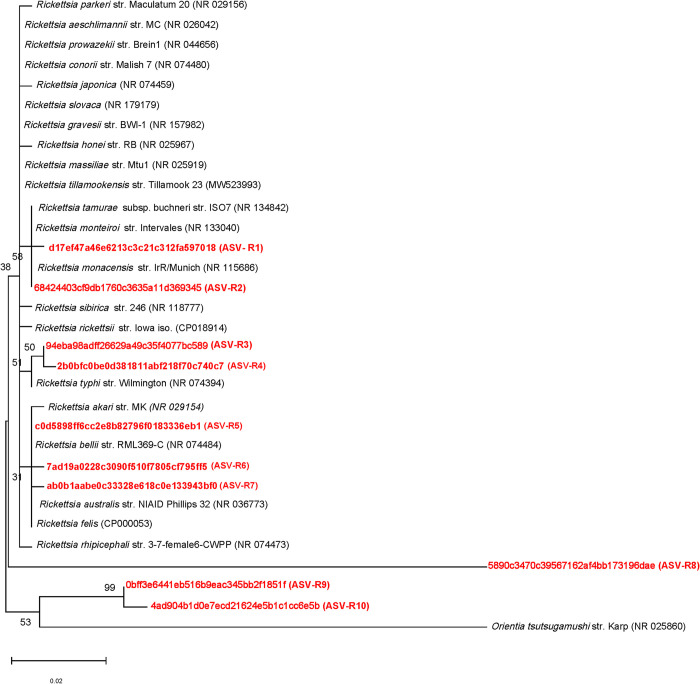
Phylogenetic analysis of *Rickettsia* amplicon sequence variants (ASVs). ASVs identified in this study (in bold red) and other known Rickettsial species (black) are shown. The phylogenetic tree was constructed by the maximum likelihood method using 16S rRNA gene sequences. The percentage of replicate trees with 50% cutoff value where the associated taxa clustered together in the bootstrap test (1000 replicates) are shown below the branches. Each ASV has an identification label in parentheses next to it. *Orientia tsutsugamushi* strain (NR025860) was included as an outgroup.

The maximum likelihood phylogenetic tree based on 400 bp V3-V4 16S rRNA sequence from *Rickettsia* amplicon sequences revealed that the *Rickettsia* detected in the chiggers were closely related to *R*. *monacensis*, *R*. *monteiroi*, *R*. *typhi*, *R*. *felis*, *R*. *belli*, and *R*. *australis* (Fig 9). ASV-R1 showed 99.75% sequence similarity to *R*. *monacensis* and *R*. *monteiroi* and 99.25% sequence similarity to *R*. *felis* and *R*. *rickettsii*. ASV-R2 was closely related to *R*. *tamurae*, *R*. *monteiroi*, and *R*. *monacensis* (100%) and 99.5% sequence similarity to *R*. *sibirica*. ASV-R3, ASV-R6 and ASV-R7 had 99.75% sequence similarity to *R*. *belli*, *R*. *felis*, and *R*. *australis*. However, ASV-R3 was also 99.75% sequence similar to *R*. *typhi* and 99.25% to *R*. *rickettsii*. ASV-R4 was closely related to *R*. *belli*, *R*. *felis*, *R*. *australis* and *R*. *typhi* at 99.50% and 99.0% to *R*. *rickettsii*. ASV-R5 was closely related to *R*. *belli*, *R*. *felis*, and *R*. *australis* with 100% sequence similarity and 99.75% to *R*. *akari*. AVS-R8 to 10, exhibited 96.10 to 89.46% homology to an *Rickettsia* spp. The pairwise distance analysis (without AVS 8–10) revealed similarity of 99.25 to 99.75% among the ASVs.

## Discussion

Chiggers are the only known vectors of *Orientia* spp., the causative agent of the disease, scrub typhus which is endemic to the Asia-Pacific regions with recent pathogenic *Orientia* species discovered in South America and the Middle East [9, 12, 13, 48]. Chiggers have also been implicated as potential vectors of a variety of other pathogens including *Rickettsia* spp., *Borrelia* spp., *Bartonella* spp., *Anaplasma* spp., and hantavirus [48]. Chiggers occur globally, and the distribution of chigger-borne pathogens is likely much larger than originally thought. Next-generation sequencing technologies, such as the amplicon sequencing method targeting the 16S rRNA gene, have revolutionized our ability to answer these disease-related questions by enabling rapid identification of emerging and re-emerging pathogens in different vectors [74, 75], including chiggers [16]. This approach has also shed light on the diverse ecological communities that exist within chiggers, providing a window into a more comprehensive understanding of chigger microbiomes [31, 48, 50]. Such knowledge is useful for preventing and identifying human diseases and offering insights into these pathogen-carrying vectors. This approach holds promise for improving the health outcomes of humans and other mammals by strengthening our prevention and identification efforts for chigger-borne diseases. Here, we characterized the bacterial microbiome, as well as potential human and animal pathogenic bacteria, from different chiggers from rodents in the southeastern US. To the best of our knowledge, our study is the first initiative to outline different chigger microbiome profiles from North America. Currently, chiggers are assumed in the USA not to vector microorganisms to humans that cause scrub typhus, Rickettesial diseases like those attributed to ticks, Lyme disease or protozoan and viral diseases.

### Chigger identification

We found that chiggers collected off rodents from Iredell, Stanly, and Craven Counties were molecularly and morphologically identified to be within the genus *Eutrombicula*. The chiggers from Rutherford County were morphologically identified to be within the genus *Leptotrombidium* but molecularly identified to have 93.85–94.23% sequence similarity to the genus *Pseudoschoengastia* and 93.08–93.46% to *Eutrombicula*. This finding implies that the chiggers from Rutherford County are likely a novel genus due to their low percent identity to known taxa. The identity of the chiggers collected in Rutheford County is still unclear but with high likelihood that it is a different species from what we have collected in the other 3 counties. Since only white-footed mice were collected in Rutherford County, this information shows that the hispid cotton rat and the white-footed mouse had different species of chiggers attached to them. These differences in chigger species are likely one of the main reasons for the microbiome differences.

### Geographic patterns of microbiome diversity and community

In this study, the comparative analysis of alpha and beta diversities revealed a significant difference in geographical boundaries and host associations that might influence the presence of chigger-associated bacteria. Environmental factors, such as habitat type, host presence, and relative humidity are known to have an effect on infection status and the microbiome in disease vectors through various mechanisms, including altered arthropod behavior and host preference [76–78]. Chiggers, in particular, are considered to have one of the most complex ecological relationships with the pathogens they vector [31, 50, 79]. For example, higher latitudinal gradients have been found to correlate with increased chigger species richness [31, 80], which in turn could affect the diversity of the microbiome. Additionally, a study that collected chiggers from small mammals in various habitat types in Malayasia, found that oil palm plantations and the surrounding habitats during the dry season had increased chigger populations as well as increased risk of scrub typhus [81]. Elliot et al. [20] found that *O*. *tsutsugamushi* infected small mammals and chiggers were positively associated with the end of the dry season, the end of the wet season, and lowland habitats. To add to this, higher relative humidity has been found to be associated with greater risk of scrub typhus infection in humans [82]. The presence and diversity of available hosts is greatly dictated by its habitat type. The host species being fed upon has been shown to significantly impact the microbiome of feeding disease vectors such as ticks. In *Ixodes scapularis* ticks, feeding on the eastern fence lizard can reduce microbiome species richness [83]. In this study, we compared the bacterial diversity of the microbiomes of chiggers collected from three different ecoregions that made up various habitat types as well as two different host species. We found the highest bacterial species richness and the highest percentage of unique ASVs in chiggers collected in Stanly County (Figs 5 and 6). Stanly County is in the eastern part of the Piedmont ecoregion and these chiggers were collected from the hispid cotton rat (S5 Fig). Iredell County chiggers, also within the Piedmont ecoregion, had the lowest number of unique ASVs (Fig 6). Chiggers collected in Rutherford County showed similar alpha diversity results to the chiggers collected in Iredell County (Figs 3 and 5). Craven County had the highest levels of alpha diversity using the metrics Faiths PD and observed features, however chiggers in this county were collected from one individual rodent (Fig 3 and Table 1).

### Bacterial composition of the chigger

There is limited information available on the microbiomes of chiggers. The microbiome of chiggers is uneven in composition, and was dominated by a limited number of highly abundant ASVs, including *Corynebacterium* (17.2%), *Propionibacterium* (12.5%), the class ZB2 (8.6%), and *Methylobacterium* (5.8%). *Corynebacterium* is a genus of gram-positive aerobic bacteria that are commonly found colonizing human skin and mucous membranes and have been detected in the microbiome of the hispid cotton rat [84]. *Propionibacterium* are a gram-positive, anaerobic, rod-shaped genus of bacteria. One of the more common species of *Propionibacterium* found in the chiggers was *Cutibacterium acnes* (formerly *Propionibacterium acnes*) which was also identified in the uninfected chigger mites in a microbiome study in Thailand [50]. A microbiome study similar to this one was conducted in Saudi Arabia, where chiggers were collected off rodents and the 16S rRNA V3-V4 region amplified [21]. This study in Saudi Arabia found *Corynebacterium*, *Mycobacterium*, *Staphylococcus*, *Candidatus* Cardinium, *Burkholderiaceae* and *Wolbachia* to be dominant in the microbiomes of all chiggers examined. Two potentially pathogenic bacterial species, *O*. *chuto* and a *Coxiella burnetii*- like organism, were discovered in the chigger species *Ericotrombidium kazeruni* and *Pentidionis agamae* [21]. Another study conducted by Chaisiri et al. investigated the microbiome of nine species of chigger collected off various small mammals in Thailand [31]. This study used similar molecular techniques, amplifying the 16s rRNA V4 region and Illumina sequencing. They found that the dominant taxa in the chiggers included: *Sphingobium*, *Mycobacterium*, Neisseriaceae and Bacillales. Human pathogens were also identified in some of these chiggers including *O*. *tsutsugamushi* and *Borrelia* spp. in L. delicense; additionally, *Staphylococcus* and *Haemophilus parainfluenzae* were found in multiple chigger species [31]. Lastly, a study on the microbiome of uninfected and infected *O*. *tsutsugamushi* in *L*. *imphalum* chigger mites found great differences in the bacterial diversity among life stages and by infection status [50]. The uninfected mites had a much greater diversity than the infected mites. Infection with *O*. *tsutsugamushi* reduced the abundance and diversity of other bacterial species and promoted mutualistic relationships with other taxa such as Amoebophilaceae [50]. The uninfected chigger mite’s bacterial composition varied greatly between life stages with the genus *Luteimonas* being the most abundant taxa for all uninfected life stages except for the larval mites. *Luteimonas* was not detected in any of the larval chiggers [50]. The chiggers analyzed in our study had a vastly different bacterial composition than the previously mentioned studies with the most similar being to the chiggers collected in Saudi Arabia [21]. It is possible that this similarity in chigger microbiome is due to collection from similar rodent hosts. The chigger microbiome is complex and diverse with factors such as the life stage, host type, the presence or absence of pathogenic species, and the environment playing a critical role in the bacterial composition.

### Presence of potentially pathogenic bacteria

Importantly, we also identified sequences with high similarity to pathogenic strains of *Orientia* spp and *Rickettsia* spp. *Rickettsia* spp. made up 0.66% of the chigger microbiome, infecting 11 (16.7%) individual chiggers among all four collection counties and collected from both host types. Four individual chiggers were found infected with *Orientia* spp. making up 0.38% of the total chigger microbiome. These *Orientia* infected chiggers were found in Stanly and Craven County, only collected off cotton rats. Recently, our group detected *Orientia* spp. in free-living *Eutrombicula* chigger mites in NC, USA, which indicates vertical circulation of *Orientia* spp. in chiggers in NC [16]. Since *Orientia* spp. were found in free-living chiggers, this finding suggests that *Orientia* spp. is being transtadially and transovarially transmitted among the life stages of these mites. These findings in combination with the findings in our study provide further molecular evidence that the potential causative agent of scrub typhus is circulating in NC, USA. However, it is not yet known how virulent the *Orientia* spp. in the USA are to mammals or if these strains may cause frank illness. Similarly, in a chigger microbiome study in Thailand that amplified the 16s rRNA gene, chiggers collected off small mammals were found infected with *O*. *tsutsugamushi* [31].

Field collected chiggers have been found infected with *Rickettsia* spp. in Thailand [85], Taiwan [28], and in NC, USA [23]. The *Rickettsia* infected field collected chiggers from the USA were from rodents in NC, and the sequences had high similarity to *R*. *japonica*, *R*. *akari*, *R*. *felis*, *R*. *conorii*, *R*. *typhi*, and other *Rickettsia* spp. [23]. These species are pathogenic to humans but are thought to be transmitted by other arthropod vectors such as ticks, mites, and fleas. This furthers the uncertainty on whether chiggers are transmitting pathogens to humans that are vectored by other arthropod vectors such as those causing tick-borne illnesses. The DNA of the chiggers used in the previously mentioned study [23] make up a subset (n = 46) of the chiggers used in this study. Ponnusamy et al. [23] used a nested PCR approach using *Rickettsia* genus primers to detect this genus in NC chiggers while the present study amplified the V3-V4 domains of the bacterial 16S rRNA gene to understand the entire microbiome. Using three different gene targets, Ponnusamy et al. [23] identified *Rickettsia* infected chiggers at the following prevalences: 47.8% (22/46) using the 23S-5S gene, 26.1% (12/46), and 15.2% (7/46). In this study, where 16S rRNA genes were amplified, we found 6 of these same 46 chiggers to be infected with *Rickettsia* spp., suggesting that *Rickettsia* were not sufficiently abundant to be detected by NGS. This is to be expected at the level of reads that were obtained per chigger and the NGS approach in amplifying and sequencing all of the bacteria in the sample. It also emphasizes the tradeoff between nested PCR and NGS for understanding the chigger infection rate for a particular bacteria genus or species. The results of this study illustrate that chiggers in the US are carrying pathogenic *Rickettsia* spp. More research is required to determine if these US chiggers are capable of spreading these Rickettsial pathogens and causing disease in humans and other mammals.

### Factors that affect the chigger microbiome

Much of the microbiome of the attached-feeding, chigger appears to be determined by what host the chigger is attached to. The environment in which that host lives could be affecting the microbial diversity and composition of the host microbiome and in turn, the chigger microbiome (Figs 4, 6, 7 and S1 and S2 Figs) [86]. In addition, the presence of specific endosymbionts can further alter the microbiome and fitness of the chigger [50, 87]. It is also possible the chigger microbiome composition is comprised of, or at least affected by, the bacteria present in the digestive system of the host they are feeding on. Further complicating this matter is the phenomenon of co-feeding of many chiggers on one animal, and the role that plays in the exchange of pathogens. It has been found that *O*. *tsutsugamushi* can be horizontally transferred from infected chigger to uninfected chigger of another species while both are feeding on the same host [42, 88]. Ticks have already shown that they are capable of spreading pathogens to other ticks through co-feeding on a shared host [89]. It is possible that chiggers are also transmitting pathogens between chiggers while co-feeding as well.

In this study, where chiggers were collected off two different common rodent host species, it was revealed that chiggers collected from the hispid cotton rat had greater bacterial diversity compared to chiggers collected off the white-footed mouse (Fig 7). Chiggers collected from the white-footed mice also had a higher abundance of *Propionibacterium* and *Corynebacterium* than chiggers collected from the hispid cotton rat (Fig 7). However, there are two limitations to interpreting these results: (i) there was a greater sample size of chiggers collected from hispid cotton rats (n = 42) than from white-footed mice (n = 24) and (ii) the white-footed mice were collected from only one collection site (Rutherford County). This observation makes it difficult to determine if the differences in bacterial composition are due to the host species microbiome or the collection location. The analysis of the bacterial abundance among collection counties revealed that Rutherford County, where the white-footed mice were collected, had the highest prevalence of *Micrococcus* (5.12%) (Figs 6 and 7), which commonly occurs in water, dust, and soil [90]. Chiggers collected from Rutherford and Iredell County had higher abundance of the genus *Corynebacterium*, *Propionibacterium*, and *Methylobacterium*, while in Stanly and Craven Counties the class ZB2 was more abundant (Fig 6). The bacterial composition of chiggers collected from Rutherford and Iredell Counties had similar results; likewise, chiggers collected from Stanly and Craven Counties shared similar bacterial composition. Rutherford County is in the Southern Appalachian Mountain ecoregion while Iredell County was in the Piedmont ecoregion. However, these two collection locations are only about 62 miles apart (Fig 1). These two collection locations differed by the species of rodent the chiggers were collected from, and the species of chigger collected. When comparing Stanly and Craven counties, these counties are over 250 miles apart and are in different ecoregions (piedmont and coastal plains, respectively), yet chiggers collected from these two sites shared a similar bacterial composition. This was an interesting result as it suggests that there are additional factors beyond spatial or ecological proximity determining the bacterial composition of the chigger microbiome.

## Conclusions and future directions

In summary, we have found (i) potentially pathogenic genera such as *Orientia* spp. and *Rickettsia* spp. in the chigger microbiome; (ii) that this microbiome is dominated by bacterial species in the genera *Corynebacterium*, *Propionibacterium*, class ZB2, and *Methylobacterium*; and (iii) that bacterial diversity and abundance differ between the host species and the collection counties. However, due to multi-variable limitations such as differing chigger species and the white-footed mouse being collected in one county, it is difficult to determine the driving source of these differences. More research is needed to discover effects of the environment on the chigger microbiome and to further understand the ecology of chigger-borne diseases.

## Supporting information

S1 Fig Alpha diversity measures of the microbiome of chiggers amongst two different hosts (Cotton rat and white-footed mouse). (A) Shannon diversity, (B) Faiths phylogenetic diversity, and (C) Observed OTUs (ASVs).
(TIF)

S2 Fig Alpha diversity measures of the microbiome of chiggers amongst eight different individual rodents (R1-R8). (A) Shannon diversity, (B) Faiths phylogenetic diversity, and (C) Observed OTUs (ASVs).
(TIF)

S3 Fig Principal coordinate analysis (PCoA) of bacterial composition in chiggers amongst two different hosts (hispid cotton rat and white-footed mouse). The analysis was based on the weighted UniFrac metric and was visualized using Emperor.
(TIF)

S4 Fig Principal coordinate analysis (PCoA) of bacterial composition in chiggers amongst eight different individual rodents (R1-R8). The analysis was based on the weighted UniFrac metric and was visualized using Emperor.
(TIF)

S5 Fig Comparison of unique and shared taxa from chiggers collected from the two host types, hispid cotton rat and white-footed mouse (WF mouse). Venn diagram showing the percentages of the common and different predicted bacterial taxa found in the microbiome of the chiggers collected off rodents in multiple counties in North Carolina, USA.
(TIF)

S6 Fig ANCOM volcano plot displaying results of the differential abundance testing among chigger collection counties. Seven ASVs differed significantly from others and were numbered in red on the figure above. These are as follows: 1-Propionibacterium (clr: 9.88, W: 338), 2-Corynebacterium (clr: 10.24, W: 350), 3- c_BD1-5 (clr: 15.01, W: 320), 4- c_ZB2 (clr: 16.02, W: 344), 5- f_Rhodobacterceae (clr: 17.71, W: 330), 6- Agrobacterium (clr: 19.16, W: 329), 7- *Candidatus Rhodoluna* (clr: 21.77, W: 334). Clr (x-axis) represents the effect of each individual feature on the entire bacterial community.The W value (y-axis) is the strength of the ANCOM test for the tested number of species.
(TIF)
