# Supplementary figures and images for: Orientia, Rickettsia, and the microbiome in rodent attached chiggers in North Carolina, USA
Source: PLoS One. 2024 Dec 5;19(12):e0311698. doi: 10.1371/journal.pone.0311698 (PMC11620566; doi:10.1371/journal.pone.0311698)

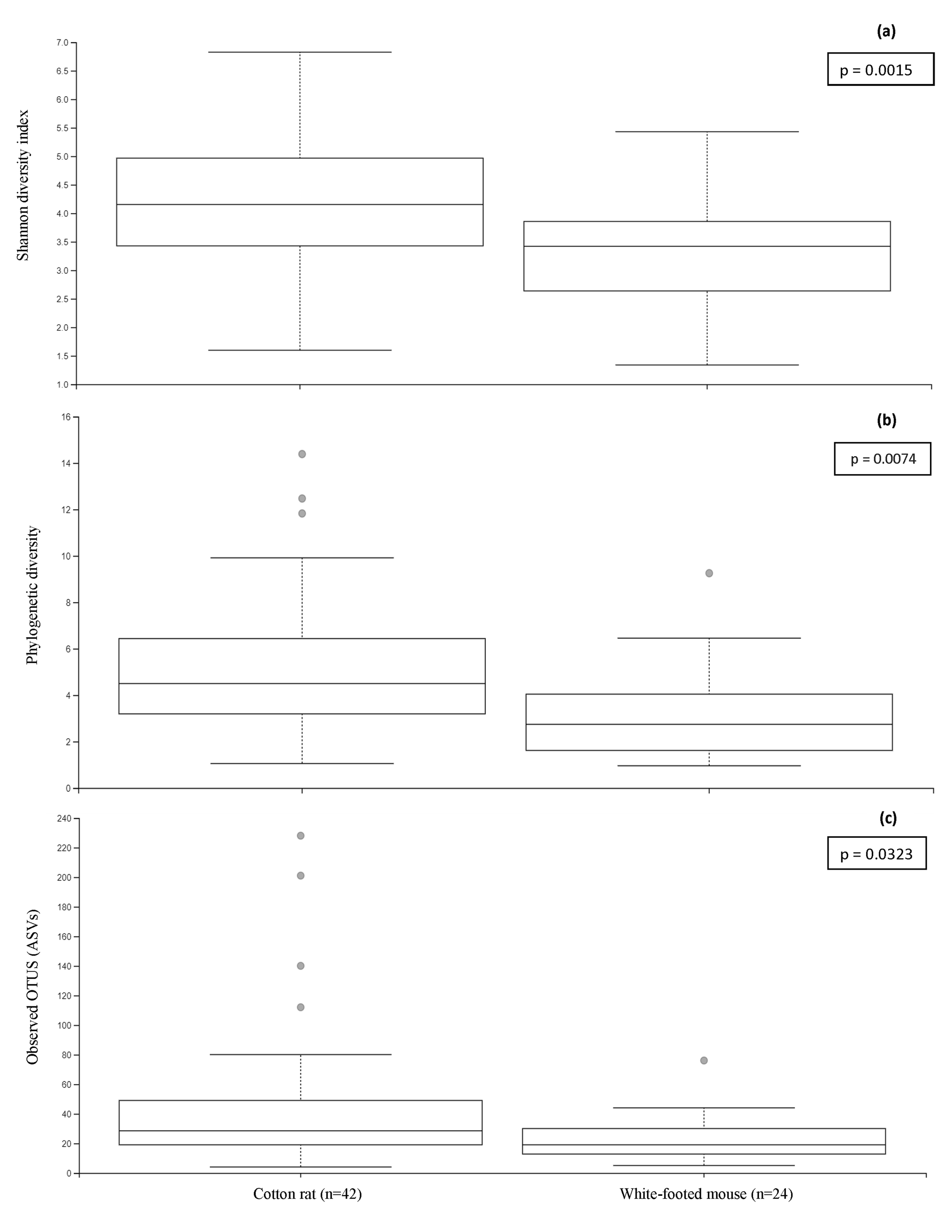

Supplement: S1 Fig — (A) Shannon diversity, (B) Faiths phylogenetic diversity, and (C) Observed OTUs (ASVs). (TIF) [file pone.0311698.s001.tif]

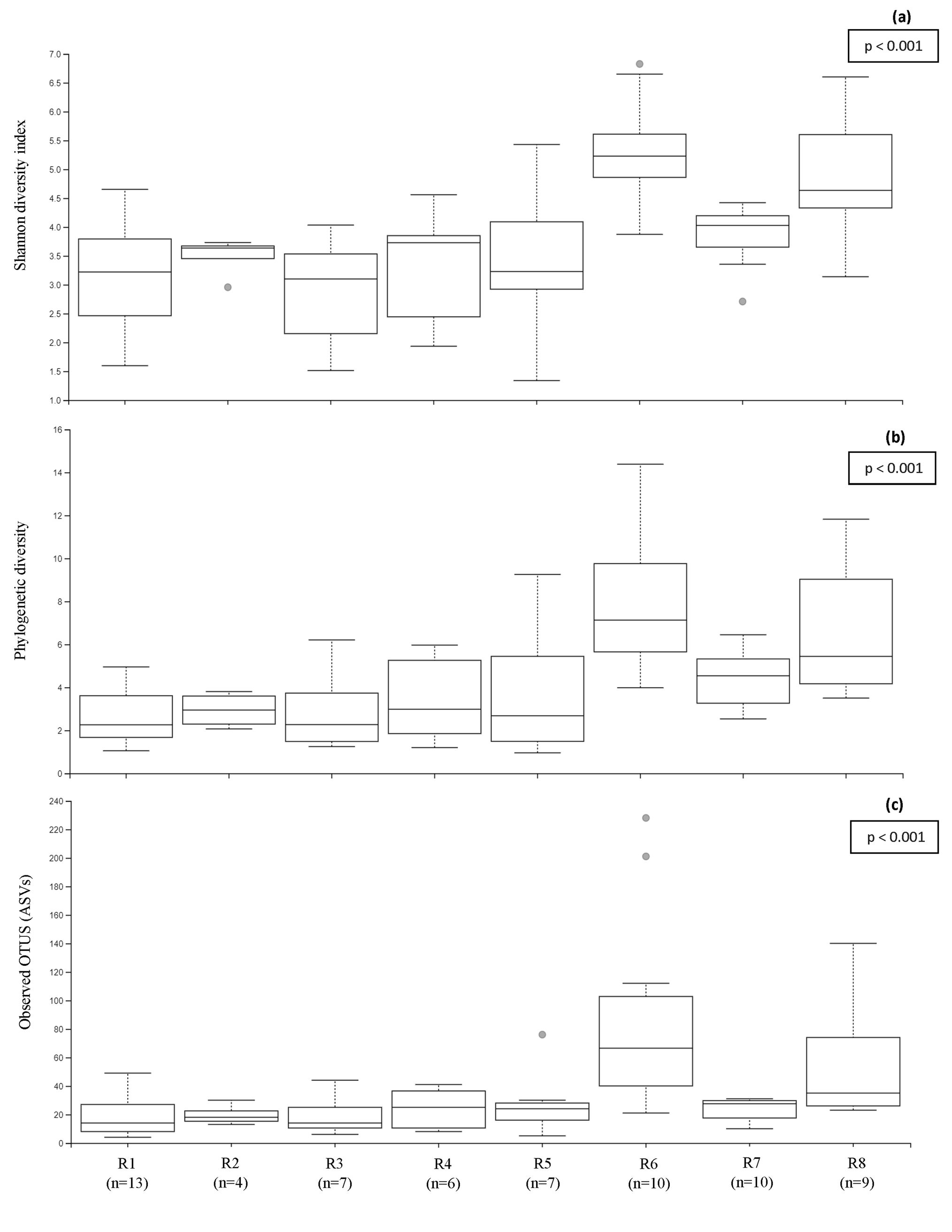

Supplement: S2 Fig — (A) Shannon diversity, (B) Faiths phylogenetic diversity, and (C) Observed OTUs (ASVs). (TIF) [file pone.0311698.s002.tif]

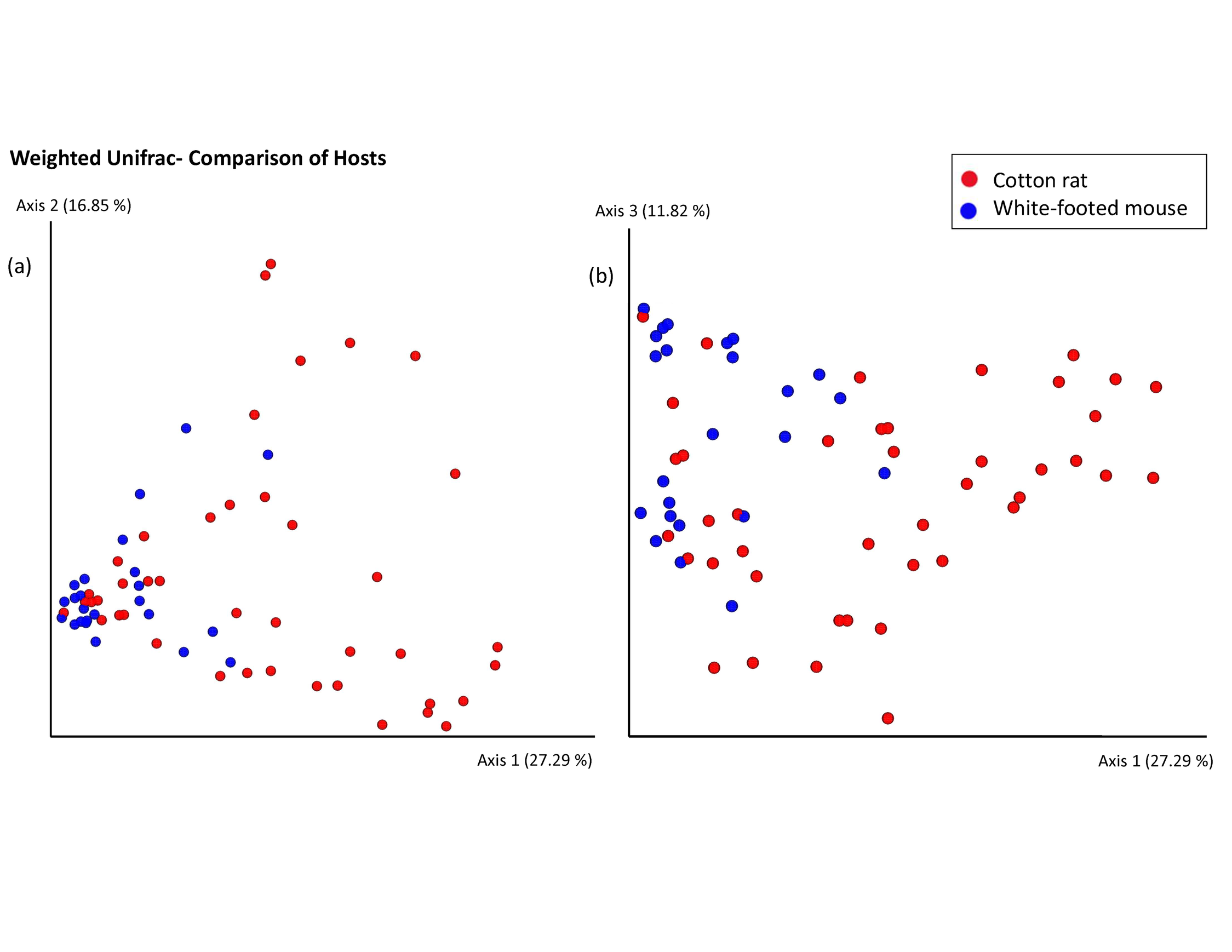

Supplement: S3 Fig — The analysis was based on the weighted UniFrac metric and was visualized using Emperor. (TIF) [file pone.0311698.s003.tif]

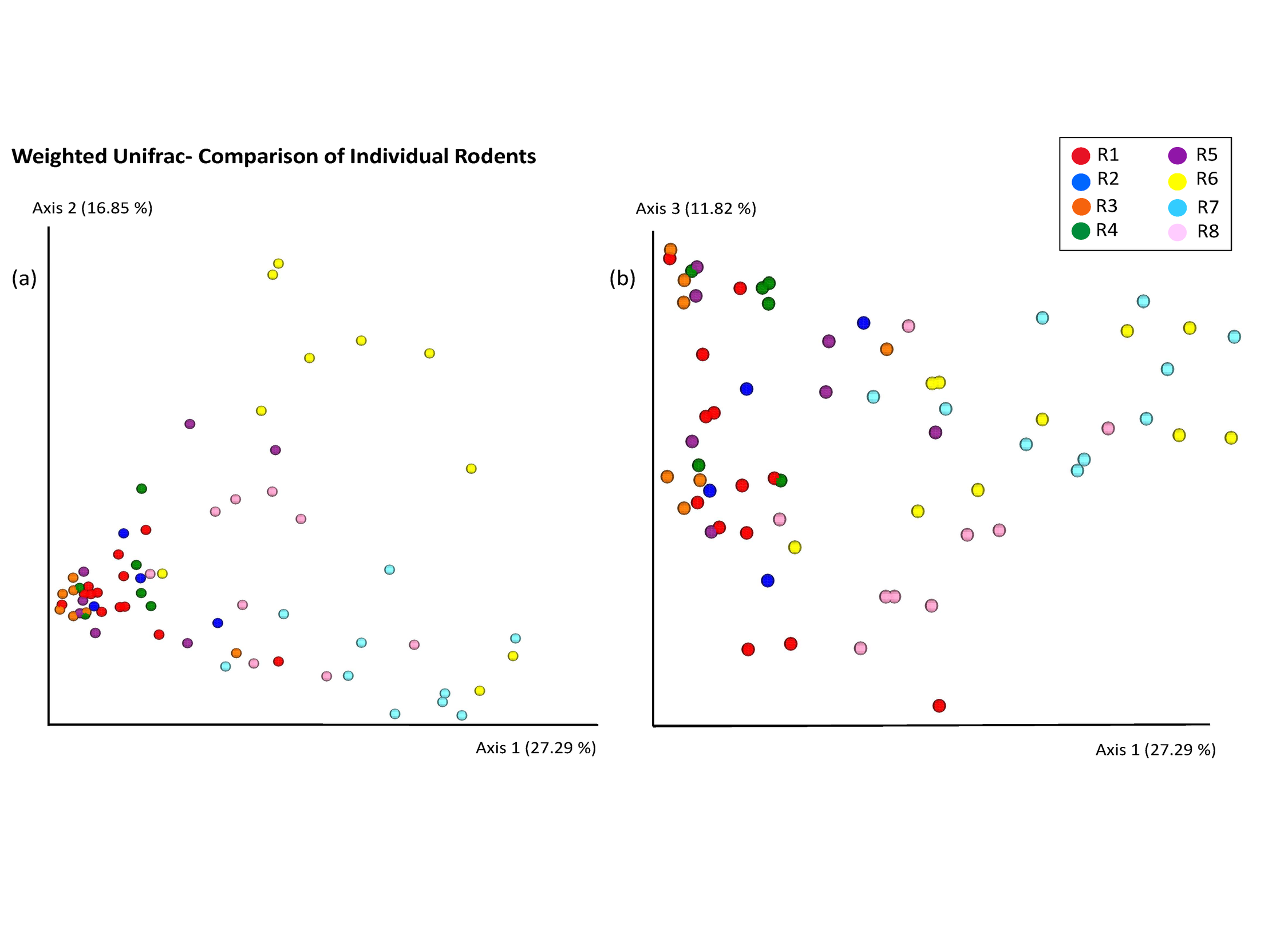

Supplement: S4 Fig — The analysis was based on the weighted UniFrac metric and was visualized using Emperor. (TIF) [file pone.0311698.s004.tif]

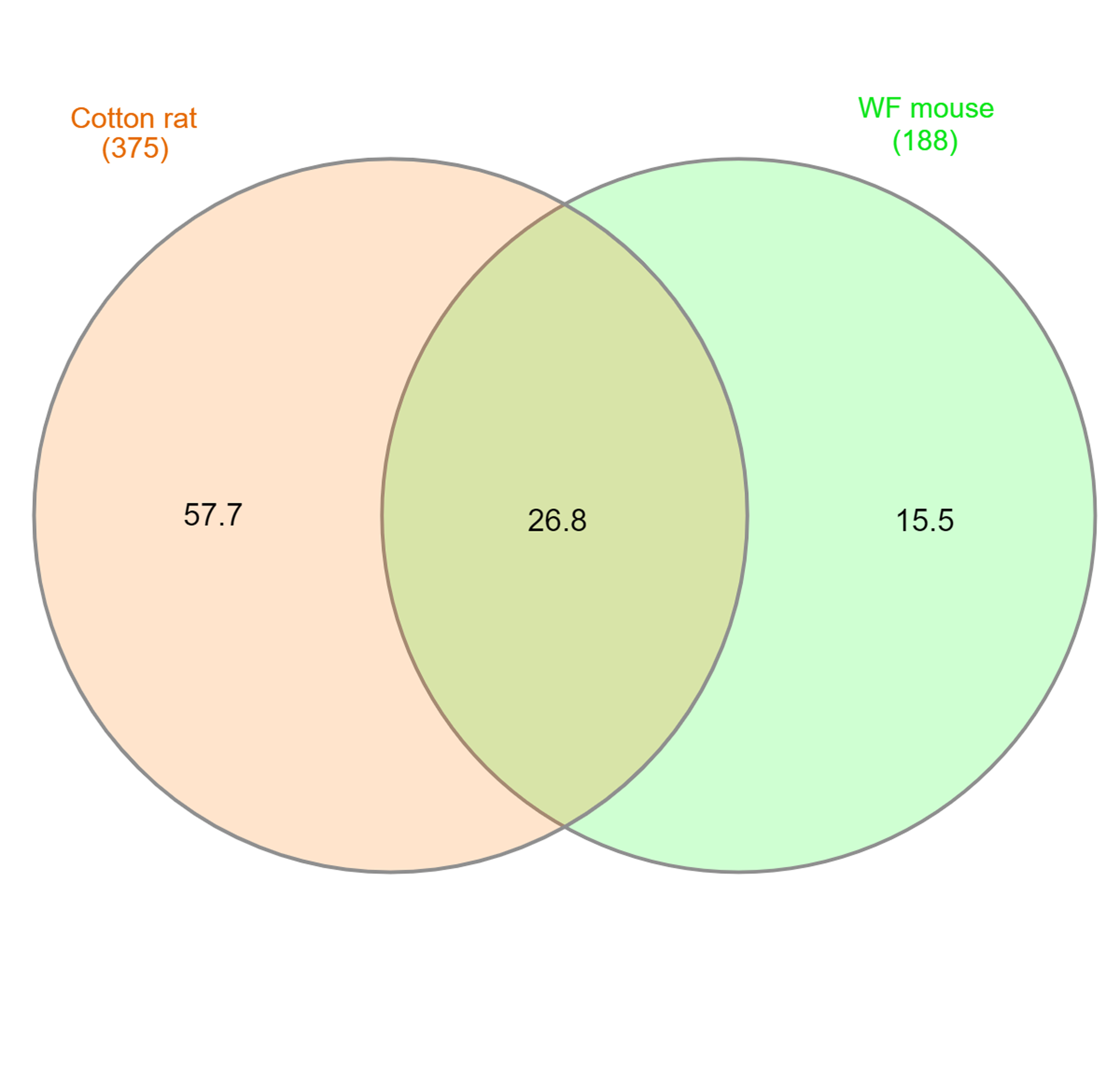

Supplement: S5 Fig — Venn diagram showing the percentages of the common and different predicted bacterial taxa found in the microbiome of the chiggers collected off rodents in multiple counties in North Carolina, USA. (TIF) [file pone.0311698.s005.tif]

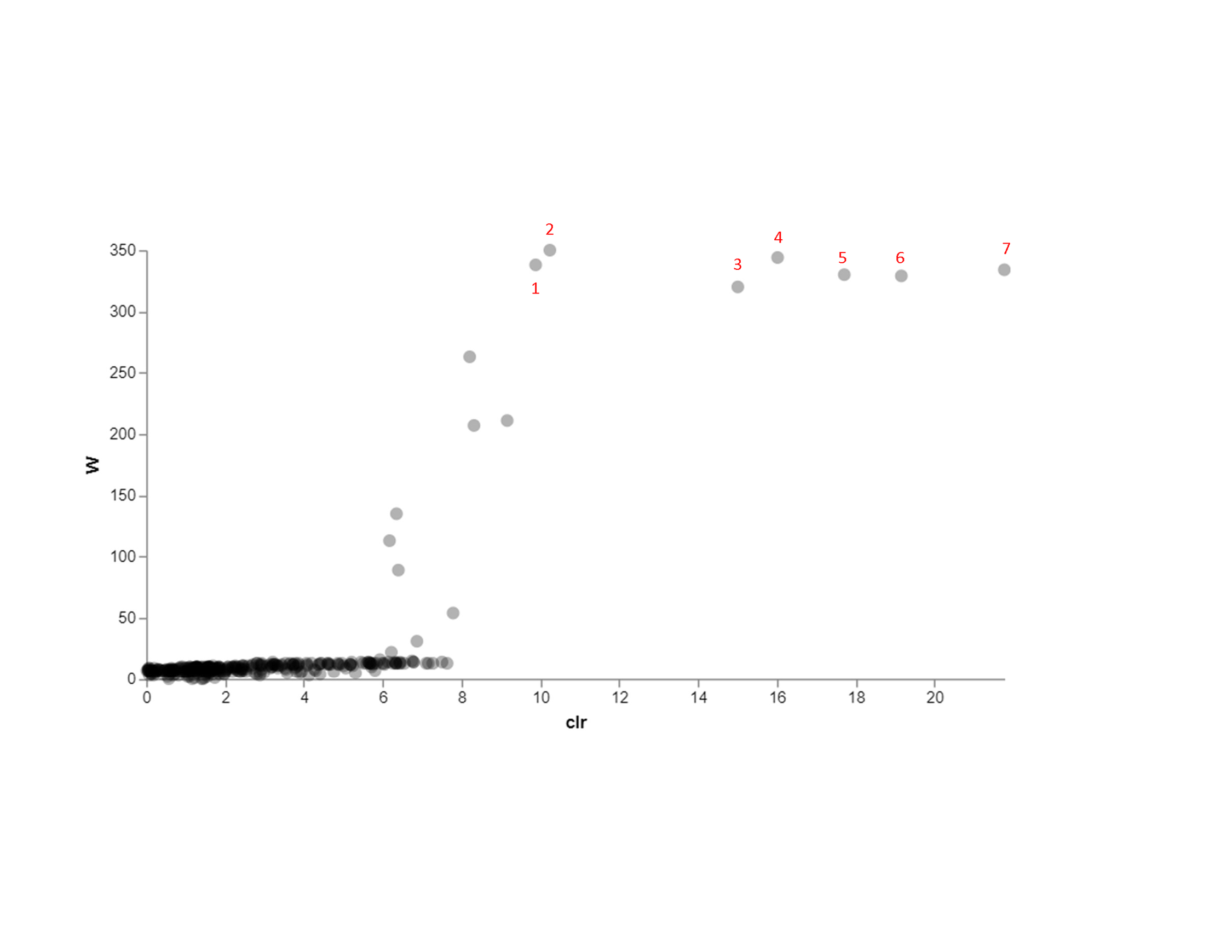

Supplement: S6 Fig — Seven ASVs differed significantly from others and were numbered in red on the figure above. These are as follows: 1-Propionibacterium (clr: 9.88, W: 338), 2-Corynebacterium (clr: 10.24, W: 350), 3- c_BD1-5 (clr: 15.01, W: 320), 4- c_ZB2 (clr: 16.02, W: 344), 5- f_Rhodobacterceae (clr: 17.71, W: 330), 6- Agrobacterium (clr: 19.16, W: 329), 7- Candidatus Rhodoluna (clr: 21.77, W: 334). Clr (x-axis) represents the effect of each individual feature on the entire bacterial community.The W value (y-axis) is the strength of the ANCOM test for the tested number of species. (TIF) [file pone.0311698.s006.tif]
